# Initial refinement of data from video‐based single‐cell tracking

**DOI:** 10.1002/cai2.88

**Published:** 2023-08-09

**Authors:** Mónica Suárez Korsnes, Reinert Korsnes

**Affiliations:** ^1^ Department of Clinical and Molecular Medicine Norwegian University of Science and Technology (NTNU) Trondheim Norway; ^2^ Korsnes Biocomputing (KoBio) Trondheim Norway

**Keywords:** big data, cancer diagnostic methods, daughter cells, phenotypic signature, single‐cell tracking

## Abstract

**Background:**

Video recording of cells offers a straightforward way to gain valuable information from their response to treatments. An indispensable step in obtaining such information involves tracking individual cells from the recorded data. A subsequent step is reducing such data to represent essential biological information. This can help to compare various single‐cell tracking data yielding a novel source of information. The vast array of potential data sources highlights the significance of methodologies prioritizing simplicity, robustness, transparency, affordability, sensor independence, and freedom from reliance on specific software or online services.

**Methods:**

The provided data presents single‐cell tracking of clonal (A549) cells as they grow in two‐dimensional (2D) monolayers over 94 hours, spanning several cell cycles. The cells are exposed to three different concentrations of yessotoxin (YTX). The data treatments showcase the parametrization of population growth curves, as well as other statistical descriptions. These include the temporal development of cell speed in family trees with and without cell death, correlations between sister cells, single‐cell average displacements, and the study of clustering tendencies.

**Results:**

Various statistics obtained from single‐cell tracking reveal patterns suitable for data compression and parametrization. These statistics encompass essential aspects such as cell division, movements, and mutual information between sister cells.

**Conclusion:**

This work presents practical examples that highlight the abundant potential information within large sets of single‐cell tracking data. Data reduction is crucial in the process of acquiring such information which can be relevant for phenotypic drug discovery and therapeutics, extending beyond standardized procedures. Conducting meaningful big data analysis typically necessitates a substantial amount of data, which can stem from standalone case studies as an initial foundation.

## Graphical abstract

This work proposes methods for data reduction to obtain essential biological information from tracking single cells in video. The primary objective is to streamline the input process for Big Data analysis, particularly when dealing with extensive databases containing vast amounts of this type of information.

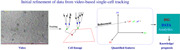

## 1 INTRODUCTION

The goal of this contribution is to showcase the potential benefits of sharing refined single‐cell tracking data obtained from video recordings. Recent advances in single‐cell research make it more interesting to follow individual cells over time to gain dynamic information from them at the individual level [1]. Data from such observations can reflect several processes and signaling pathways inside cells and between them. Tracking single cells in video aspires to provide this type of information, which can already be lost when working on fixed dead cells. Such tracking can also contribute to characterize phenotypic states and quantify them, as permanent or temporary [2, 3]. It can provide data on lineage relationships between cells and their descendants, contributing to trace population dynamics and insight into possible pathological outcomes [4].

Single‐cell tracking is especially relevant for studying cancer cells, which are known to exhibit highly adaptable behavior during treatments. Cancer cells can rapidly alter their gene expression profiles to adapt to new microenvironments, making them difficult to target effectively [5]. This high plasticity also enables cancer cells to fuse during close cellular interactions, generating hybrid subpopulations with enhanced tumorigenicity and metastatic capacity [6, 7, 8]. In addition, cancer cells can display significant phenotypic heterogeneity within genetically identical populations as a result of unique transcriptomes and proteomes [9]. This heterogeneity, which can be driven by epigenetic alterations, poses a challenge for guiding personalized treatments [10, 11, 12, 13]. Tracking single cells over time can provide valuable insights into lineage relationships and population dynamics, shedding light on the mechanisms behind these phenomena.

Several authors emphasize that single‐cell tracking from video has broadened the spectrum in mammalian signaling networks, drug development, and cancer research [14, 15, 16, 17, 18, 19, 20, 21, 22, 23]. Refs. [19, 24, 25] showed statistics from systematic single‐cell tracking during several days, elucidating heterogeneous cell response and induction of cell death mechanisms. This tracking also allowed detection of inheritable traits, such as vacuolar transfer from mother to daughter cells. Inheritance may here be significant for the interpretation of observations related to autophagy signaling [26, 27].

Andrei et al. [28] pointed out different types of observables from tracking two‐dimensional (2D) cell cultures that might have biological relevance in cellular studies. 2D cultures have provided a wealth of information on fundamental biological processes and diseases over the past decades [29]. The advantage of using these models for tracking single cells is their low cost and reproducibility as compared to three‐dimensional (3D) platforms [30, 31, 32]. 2D models can easily integrate subsequent biochemical analysis and act as surrogate measurements for 3D situations [33].

3D models are under active development to better represent the complexity of living organisms during in vitro research [34, 35, 36, 37]. However, they still do not recapitulate micro‐environmental factors, being only reductionist of the in vivo counterpart [29, 33]. 3D cell culture models are currently application specific and experiments with them are difficult to check for repeatability [29]. Current 3D platforms do not allow acquisition of cellular kinetics with a high spatial and temporal resolution over a long period of time [33, 38]. High‐content screening (HCS) platforms are emerging, however, visualization of 3D structures growing within complex geometrical structures remain still a big challenge mainly due to optical light scattering, light absorption, and poor light penetration with prolonged imaging acquisition times [29]. Microfluidic devices under highly controllable environmental conditions is a well‐established operation in ongoing research [39]. However, optimal nutrient supply and sufficient cell retention, especially for the long‐term cultivation of slow‐growing cells as well as motile cells, still requires a reliable cell retention concept to prevent permanent cell loss, which otherwise compromises qualitative and quantitative cell studies [40].

This study restricts to processing data from tracking individual cells growing in 2D monolayers. The intention is to show, by simple examples, the potential utility of large collections of such data, allowing users to compare their experiments with many previous similar experiments. Such collections would facilitate big data analysis, taking advantage of weak correlations in large amounts of data. The source of these data may be video recordings of diverse quality, assumed as by‐products from experiments worldwide. The present example data therefore, for the sake of simplicity, only represent positions (tracks) of individual cells and their eventual division and death during recording. They originate from previous work on Yessotoxin (YTX) [19]. This small molecule compound can induce different cell death modalities [41]. The broad spectrum of cellular response to YTX suits the present illustrations. The richness of responses from it may also make the compound an interesting candidate to probe cells for properties.

Data collections from single‐cell tracking can be a resource for both experimental work and statistical investigations, including fault‐tolerant big data analysis to search for patterns of biologic relevance. The present data processing may also have direct interest for processing videos aimed at special studies on possible emergence of rare or resistant subpopulations among cells subject to toxic agents, potential for metastasis or early screening for drug discovery. Another actual application is simply to check for the healthiness of cell populations, including testing for contamination.

The data analyses below relates cells in pedigree trees, where the initial cells are the ancestors (roots). These trees facilitate classification of cells in subpopulations according to a combined analysis of the cells in each tree. An example of such a combined analysis is to count the number of dying cells in each pedigree tree. The statistics below apply this simple idea assuming that cells in pedigree trees, with no cell death, might define a special resistant subpopulation. It reflects, for different subpopulations, variation in cell speed, correlations between sister cells as well as relocation and tendency of clustering. The authors conjecture that such data summaries can guide computerized search after patterns and causal relations in large sets of single‐cell tracking data. The final proof of concept depends on access to such data sets.

A variety of relatively low‐cost equipment apply to perform video‐based single‐cell tracking in 2D cellular models. Researchers can now in their most cost‐effective way produce videos of living cells for subsequent analysis by remote (Internet/cloud based) services, as recently developed by Korsnes Biocomputing (KoBio).^1^ They may also do similar analysis/tracking using their own favorite tools, such as Image J/TrackMate [42]. The supplementary data illustrates the potential transparency and software/equipment independence of such data production^2^ facilitating sample inspection. Perturbation of data values can reveal if analysis results are sensitive to measurement errors. These factors make such data relevant for contribution to biological databases reviewed by Zou et al. [43], and Haniﬀa et al., [44, 45]. The main intention here is taking advantage to utilize data from simple and low‐cost recordings to create synergistic value from sharing data on cellular behavior.

## 2 MATERIALS AND METHODS

### 2.1 Toxin

YTX was obtained from the Cawthron Institute (Nelson, New Zealand). YTX was dissolved in methanol as a 50 µM stock solution. The stock solution was diluted in RPMI medium (Lonza, Norway), achieving a final concentration of 2 µM YTX in 0.2% methanol. Treated cells were incubated with 200, 500, and 1000 nM YTX and control cells were incubated with 0.2% methanol as vehicle.

### 2.2 Cell culture

A549 cell lines were provided by Dr. Yvonne Andersson and Dr. Gunhild Mari Mœlandsmo from the Institute of Cancer Research at the Norwegian Radium Hospital. Cells were cultured in RPMI 1640 (Lonza, Norway), supplemented with 9% heat‐inactivated fetal calf serum (FCS, Bionordika), 0.02 M Hepes buffer 1 M in 0.85% NaCl (Cambrex no 0750, #BE17‐737G) and 10 mL 1X Glutamax (100X, Gibco #35050‐038), 5 mL in 500 mL medium. Cells were maintained at 37°C in a humidified 5% CO_2_ atmosphere.

### 2.3 Single live‐cell imaging and tracking

A549 cells were plated onto 96 multiwell black microplates (Greiner Bio‐One GmbH) for time‐lapse imaging. Cells were imaged into Cytation 5 Cell Imaging Reader (Biotek), with temperature and gas control set to 37°C and 5% CO_2_ atmosphere, respectively. Sequential imaging of each well was taken using a 10x objective.

The bright and phase contrast imaging channel was used for image recording. Two times, two partly overlapping images were stitched together to form images of the appropriate size. A continuous kinetic procedure was chosen where imaging was carried out with each designated well within an interval of 6 min for a 94 h incubation period. Exposed cells were recorded simultaneously subject to three different concentrations of YTX 200, 500, and 1000 nM.

The single‐cell tracking in this work was performed using the in‐house computer program Kobio Celltrack.^3^ The present data derives from previous work on YTX [19].

## 3 RESULTS

### 3.1 Single‐cell tracking

**Figure 1:**
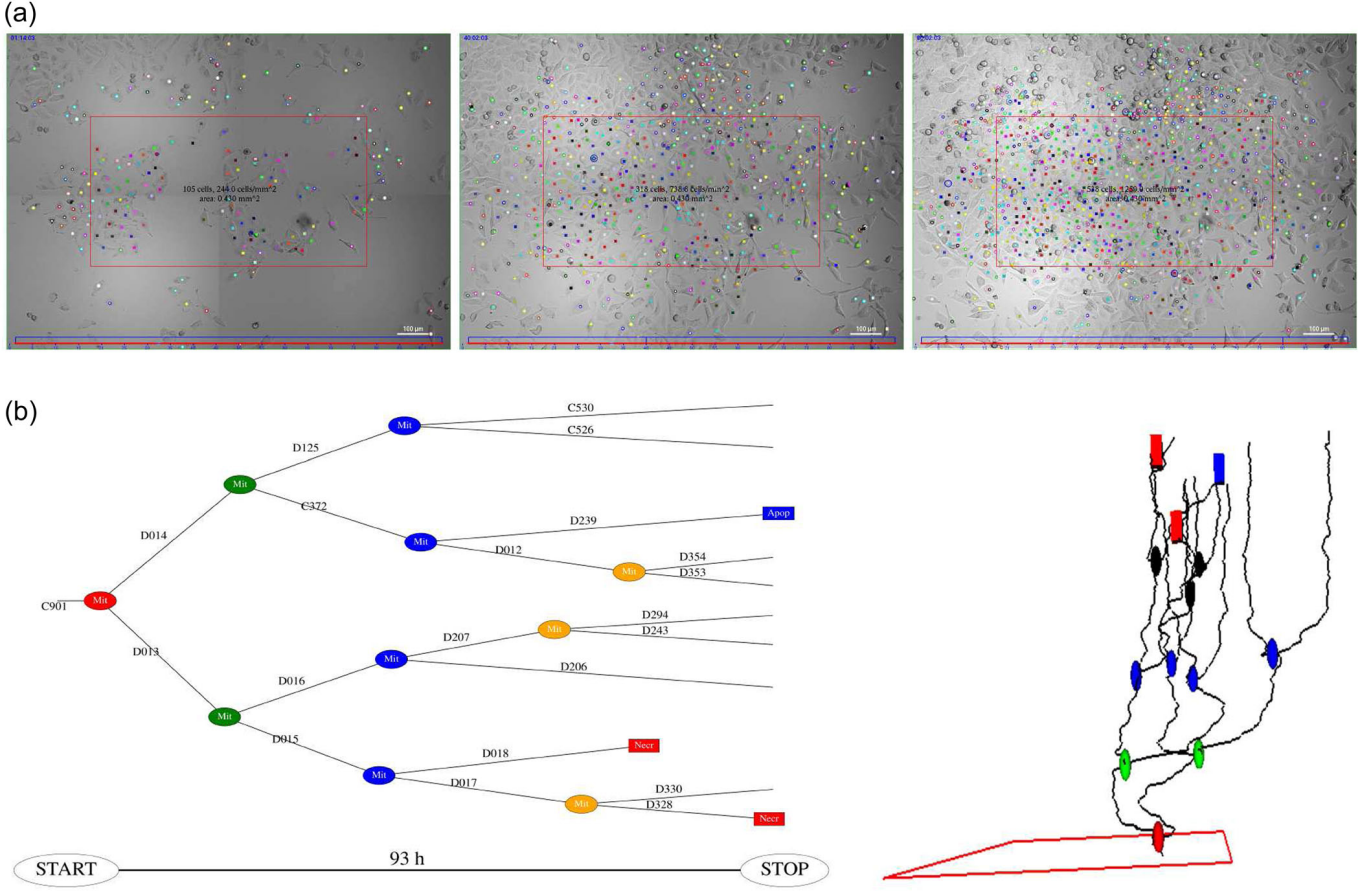
Illustration of production of single‐cell tracking data for subsequent analysis and data compression aimed for big data analysis. Cells were in this case exposed to 200 nM Yessotoxin (YTX). The supplementary data includes video demonstrations of the actual tracking (see footnote 2). (a) Snapshots illustrating tracking individual cells from video of A549 lung cancer cells. Left: at the start, center: after 40 h, right: 80 h. The actual recording instrument was Cytation 5 with 10× magnification. Each image consists of 2 × 2 stitched (approximately) simultaneous images. The red frame is just large enough to contain 100 cells at start of recording. All these 100 cells and their descendants are subjected to subsequent tracking (see supplementary data). (b) Left: “flat” temporal representation of a pedigree tree showing cell tags/names for reference in communications. Cell division appears as ovals, where their color depends on generation. Rectangles represent cell death (blue: apoptosis‐like, red: necrosis‐like). Right: 3D illustration of the same pedigree tree, providing information on motion of the cells.

Figure 1 illustrates production of input data for the present analysis. The colored dots in Figure 1a represent individual cell positions during 94 h of recording. The tracking also provides data on cell division and death. The actual tools for tracking are outside the scope of this study, which in principle could rely on data from any functional tracking system.

Figure 1b illustrates data products from the prior single‐cell tracking. The left part of the figure gives a time‐attributed graph representation of kinships between the descendants of a cell which is inside the red frame at start of recording. The right part illustrates the positions of these cells during recording. The horizontal positions (*x‐y* coordinates) here represent spatial location and the height (*z*‐coordinate) represents time. The red frame is here just large enough to contain 100 root cells at the start of recording. The present examples of statistical analysis are for the cells belonging to the pedigree trees starting inside such a red frame.

**Figure 2:**
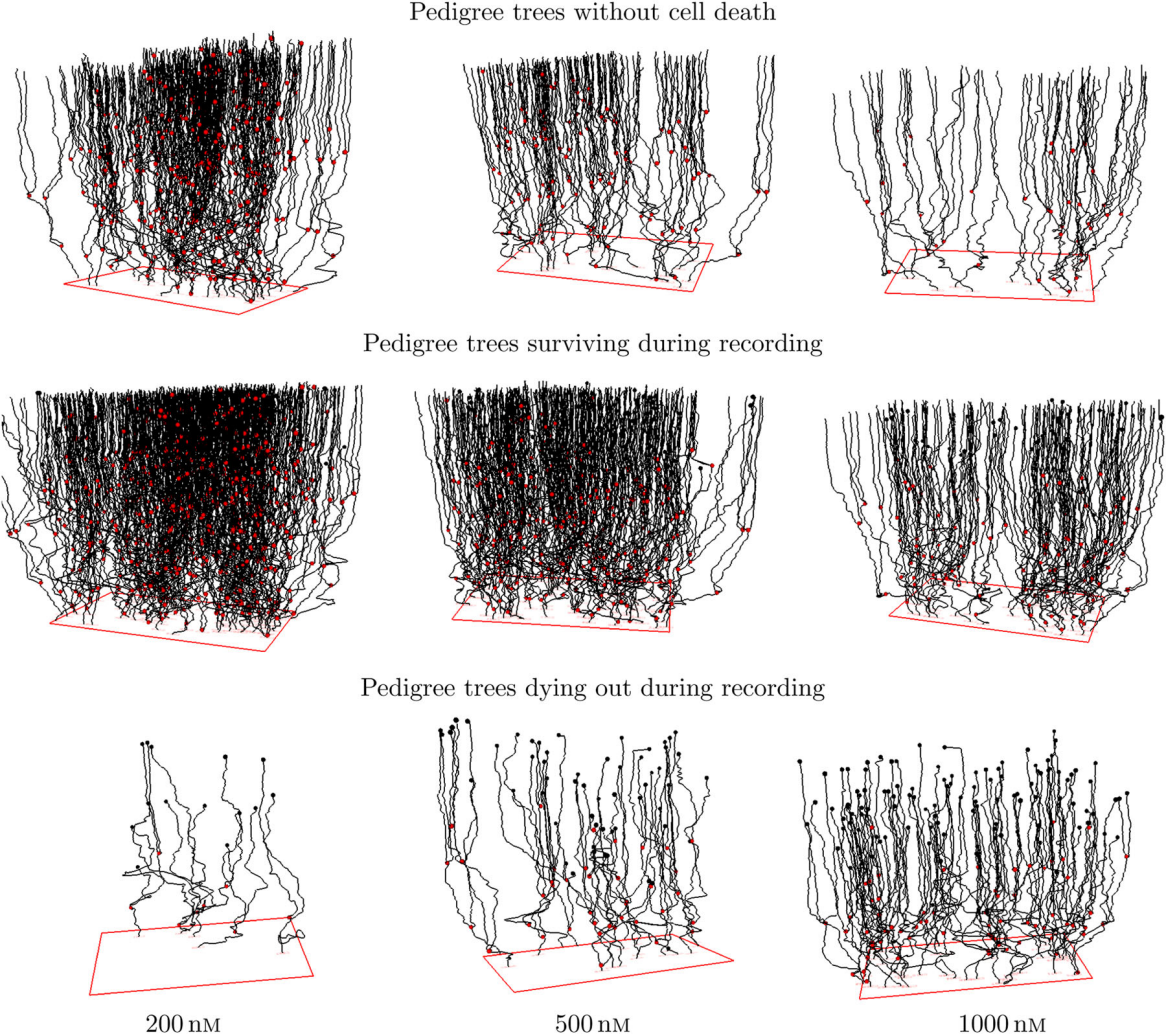
“Forest” of pedigree trees from tracking A549 cells exposed to Yessotoxin (YTX) at concentrations 200, 500, and 1000 nM. The upper row shows trajectories for cells in lineages without death (“resistant cells”). The middle row is for trajectories of cells in lineages where at least one cell lives at the end of recording (“surviving pedigree trees”). The lower row shows trajectories for cells in lineages dying out during recording. Red and black dots represent cell division and cell death respectively. Note that single‐cell tracking can provide more precise information on cell viability as compared to traditional bulk assays. These types of measurements are prone to overestimate cell survival due to prior apoptotic cell clearance and disintegration.

Figure 2 shows spatially located pedigree trees for cells exposed to YTX at three different concentrations. Cells in surviving lineages exposed to the highest YTX concentration (1000 nM) may appear to behave similar to cells subject to the lowest YTX (200 nM) concentration. It could reflect a resistant subpopulation.

### 3.2 Single‐cell viability

Cell tracking offers valuable insights into fundamental cellular properties like survival and proliferation, making it a crucial tool across various domains of cell research such as risk assessment for toxic agents, drug screening, and cancer research. Researchers studying the impact of specific toxic agents on a particular group of cells can enhance their understanding by comparing their findings with data from similar experiments conducted elsewhere. Efficient reduction of such data plays a pivotal role in facilitating meaningful comparisons and enabling access to relevant information within extensive data collections. This section presents prototypes of data reduction techniques aimed at achieving these objectives.

**Figure 3:**
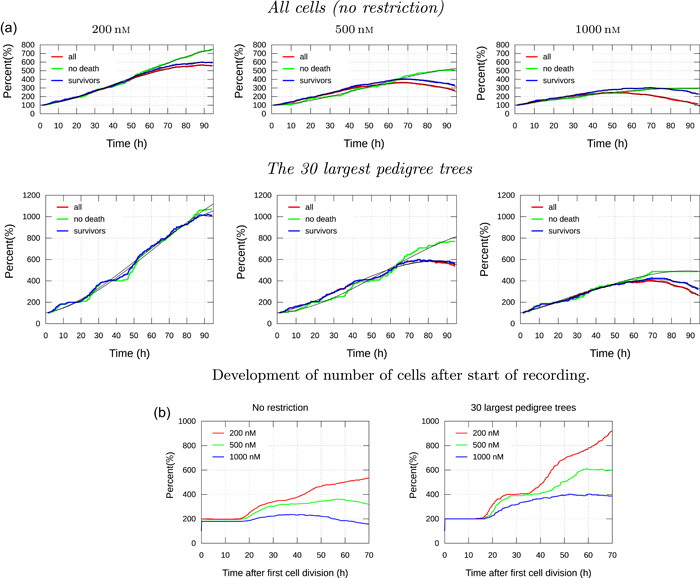
Illustration of different views of cell proliferation for A549 cells exposed to 200, 500, and 1000 nM Yessotoxin (YTX) concentrations. Note that “all” refers to all cells in the red frame (see Figure 1a); “no death”: cells in lineages with no death; “survivors”: cells in pedigree trees where at least one cell lives at the end of recording. Note the smoothness of the graphs, enabling effective parametrization (“data compression”). (a) Development of the number of cells after the start of recording. (b) Development of the number of cells after the first cell division. The graphs start at 200%, reflecting the doubling of the number of cells immediately after the first cell division.

**Figure 4:**
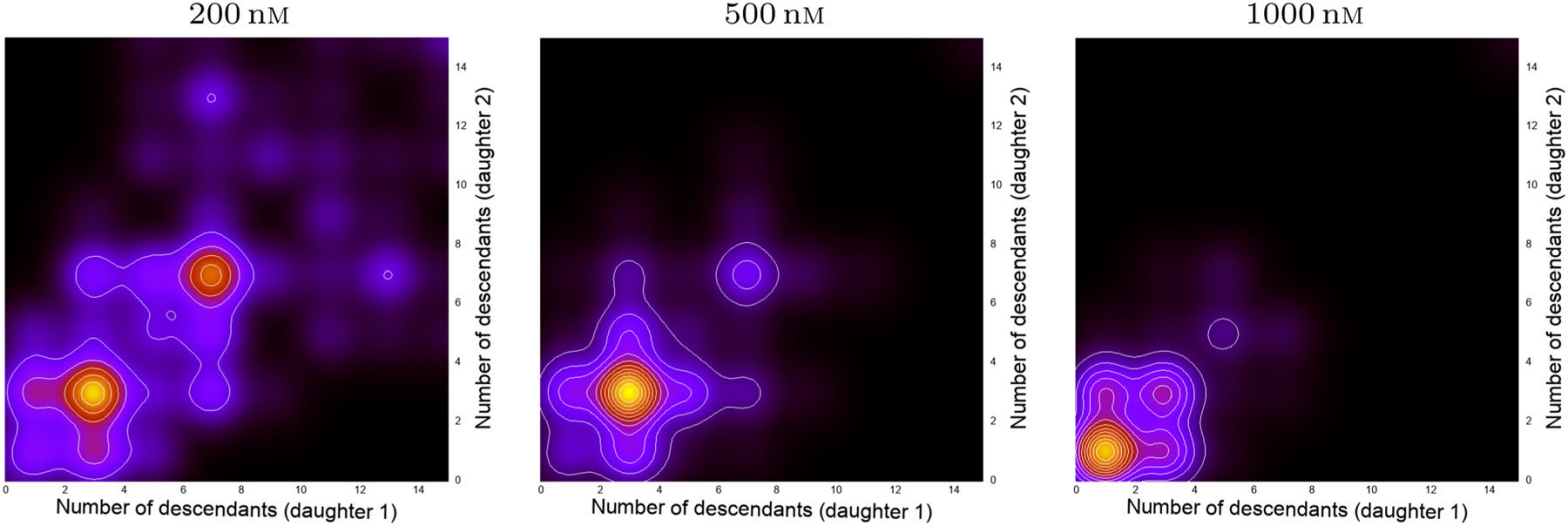
Results from kernel smoothing (bandwidth 1.5) of stack plots for number of descendants of first‐generation sister cells within 70 h after their birth. The plots are for cells born within 20 h after start of recording. The two apparent clusters in the plot for cells exposed to 200 nM Yessotoxin (YTX) indicate inheritance from the common mother cell.

Figure 3a shows the change in the size of distinct cell subpopulations during video recording. The graphs show the development of number of cells in pedigree trees with roots (initial ancestors) inside a frame centered in the video and just large enough to contain 100 cells at the start of recording. The population of cells belonging to the largest pedigree trees naturally grows faster than the total population. These cells potentially dominate in number after some time, if they inherit their tendency of cell division and survival. Correlations between proliferation and survival of descendants of sister cells (see Figure 4) can indicate such inheritance.

The lower row in Figure 3a illustrates that cell “viability analysis” based on single‐cell tracking can provide information beyond results from traditional bulk analysis. The black solid lines in Figure 3a represent a third‐degree polynomial model fit to the data:

(1) $$P_{3}\;\left(t\right)=100+at+bt^{2}+ct^{3},$$

where *a*, *b*, and *c* are the (model) parameters and t represents time. Polynomials (or Taylor expansions) are generally a convenient way to represent smooth (“simple”) functions and to compress data (representing it by three parameters). Parameters from fitting a complex biologically justified model may not necessarily represent more biologically relevant information if they are less effective to compress data.

Assume fitting a Taylor model (Equation 1) to the data as above (see Figure 3a). Consider the resulting parameters as a point, **P** = (*a*, *b*, *c*), in the three‐dimensional parameter space. Similar parameters from various experiments will give a set of points in the parameter space. If these points spread out close to, for example, a 2D structure (embedded in the 3D space), then there should, intuitively, be hope for finding statistical models with two parameters (instead of three) providing a biological interpretation/understanding. Voids in the parameter space can also represent knowledge.

Figure 3b shows the percentage development of the number of cells in pedigree trees as a function of time after the first cell division. The left part of the figure is for all 100 pedigree tress (initiating in the red frame as explained above), and the right part is for the 30 largest pedigree trees. The figure shows that cells exposed to the lowest concentration of YTX (200 nM) tend to follow a regular timing for cell division, as opposed to those subject to the highest concentration (1000 nM). This tendency is most expressed for the largest pedigree trees (right part of the figure).

### 3.3 Speed

Measurements of cell speed offer valuable insights into cellular conditions following various treatments. This information holds significant prognostic value by providing indications of cellular response and potential outcomes associated with specific interventions. For instance, it can contribute to the identification of distinct migration and persistence values that may correlate with the rate of intravasation [46].

Similar arguments for data reductions of viability, discussed in Section 3.2, are also applicable to cell speed. It is worth noting that viability and speed are likely to be correlated, which presents additional opportunities for data reduction, including dimensionality reduction techniques [47].

Track length for a cell during a period of time *t* (divided by *t*) can intuitively define its average speed during that period. However, track length is not in practice directly available nor be it well‐defined for imprecise and irregular positional data, where measures of length can depend on resolution. Cell speed could (ad hoc) refer to movements of a given defined point in a cell (e.g., the mean point of the nucleus/nuclei). However, it may principally be looked at as a spatio‐temporally localized (statistical) property of a cell. Future work may assume an “uncertainty principle” where a positional data point is considered a random selection from a set of possible positions depending on the tracking method. An alternative approach is to increase the level of sophistication and replace the concept of “cell speed” with temporal change in the (segmented) set of points covered by an actual cell.

Estimates of positions are, for any definition, imprecise for low‐quality imagery data. This work, therefore, for the sake of simplicity, demonstrates Gaussian kernel smoothing and interpolation [48] to define speed. The actual bandwidth is 15 min. Perturbations of estimates of cell positions may help to reveal how final results are sensitive to this choice of bandwidth. The authors left  this exercise as a separate study. Note that big data approaches may in principle automatically sort out useful definitions of speed.

**Figure 5:**
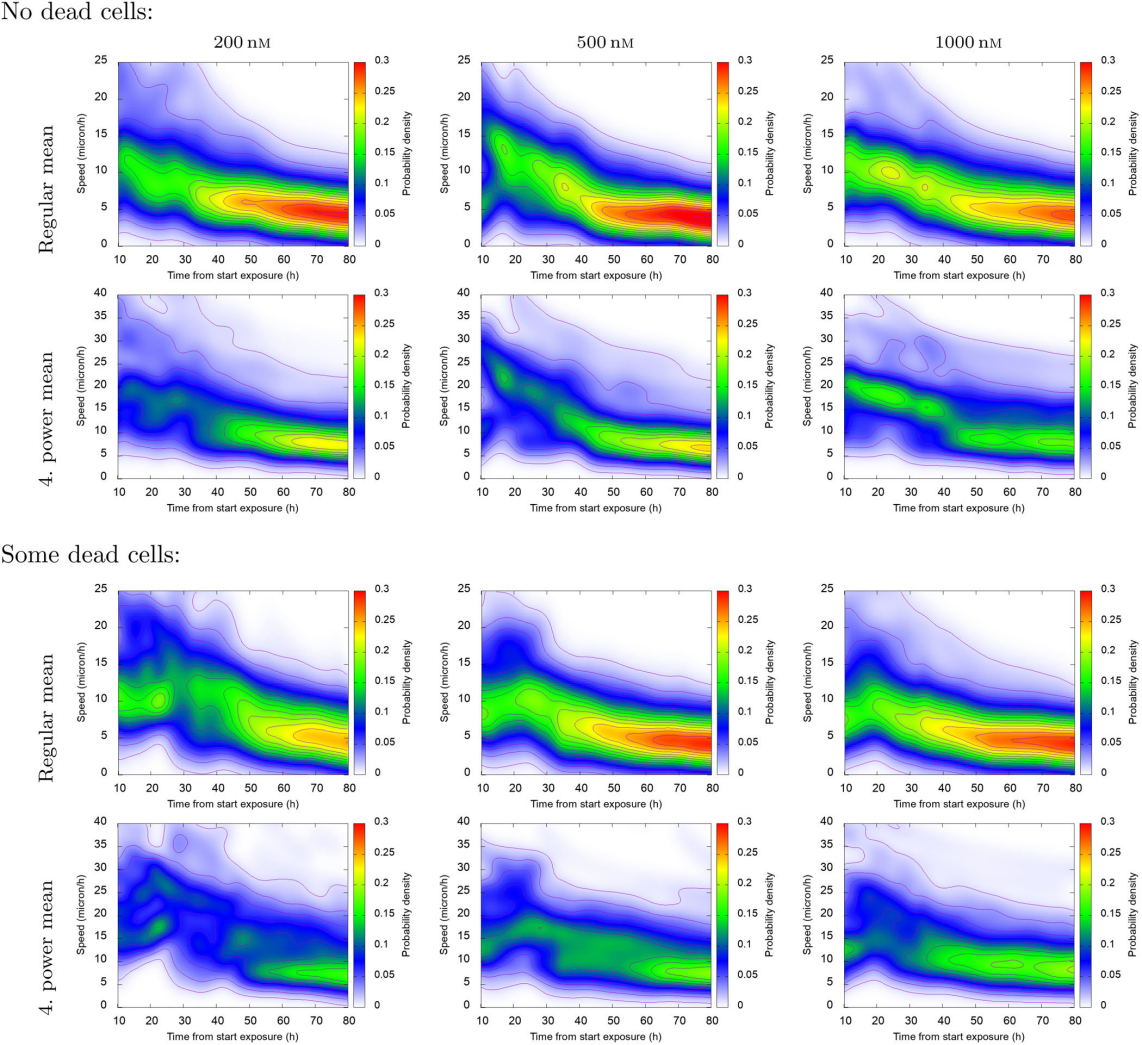
Distribution of 8 h centered running generalized mean of speed of cells during recording. The top and third row show regular (first order) mean, and the second and fourth row show fourth power mean (*M_p_*, *p* = 4). Note the difference between the distributions, especially at the first part of the recording.

Figure 5 shows distributions of the 8 h centered moving generalized mean speed for cells in lineages with and without death during recording. The upper and third rows are for the regular mean, whereas the second and lower rows similarly show the fourth power mean for the same data. This example illustrates a possible data product that presumably could provide information to big data analysis. The power mean *M_p_* is increasingly more sensitive to the highest speeds for increased values of *p*. The distribution for *M*_4_, for example, seems to be more sensitive to cell death in lineages as compared to lineages with no cell death. One can expect that the power mean *M_p_* for *p* = 1, 2, …, *n* will in a compact way reflect the distribution of speed for a restricted value of *n*.

### 3.4 Correlation between descendants of sister cells

Correlating or analyzing the mutual information^4^ between parameters of sister cells can reveal signaling downstream lineages. The treatment of cells can affect their signaling and potentially introduce noise during cell division affecting the behavior of descendant cells. As a result, single‐cell tracking data has the potential to capture and reflect this valuable information. When multiple cell types exhibit similar responses to similar treatments performed at different laboratories, they can provide deeper insights into cellular reactions. By comparing single‐cell tracking data from different experiments, we can facilitate the discovery of robust findings. This section outlines ideas for summarizing or reducing the data to facilitate this search.

**Figure 6:**
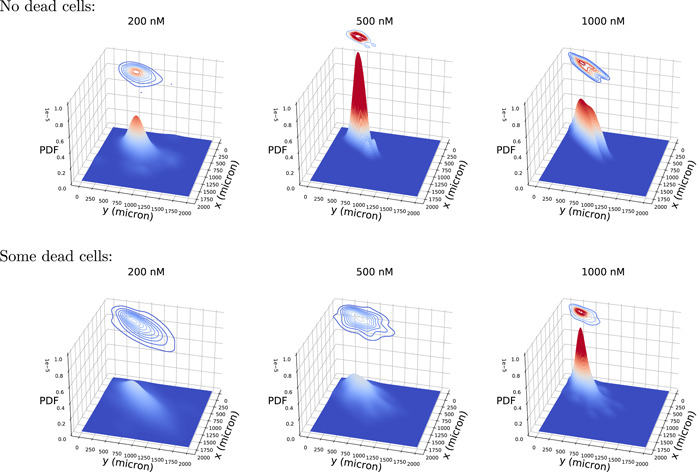
Joint probability density function (PDF) of total track length (*x* and *y*) for the first generation sister cells and their descendants 60 h after the birth of these (initial) sister cells. The cells are subject to YTX exposure at concentrations 200 nm, 500 nm and 1000 nm. The upper row shows distributions for the pedigree trees with no cell death, and the lower one shows pedigree trees with some cell death.

Figure 6 shows joint distributions for the total track length of first‐generation sister cells and their descendants 60 h after the birth of these (initial) sister cells. These statistics are restricted to sister cells born within 30 h after start of the recording. The estimates result from using the algorithm scipy.stats.gaussian_kde from SciPy^5^ with default settings (i.e., the “scott” method defines the estimator bandwidth). Section 3.3 outlines the present estimation of length from imprecise positional data (applying Gaussian kernel smoothing).

The joint distributions of Figure 6 show positive correlations and hence reflect inheritance from mother cells to their daughters. The authors will not further speculate on the biological significance of these statistics, since they only reflect results from one experiment. However, the main finding here is that such distributions are sensitive to cell treatment. One may therefore suspect such data summaries to be relevant for big data analysis. The regularity of such distributions enables effective parametrization (or data compression) to help search in large databases.

**Figure 7:**
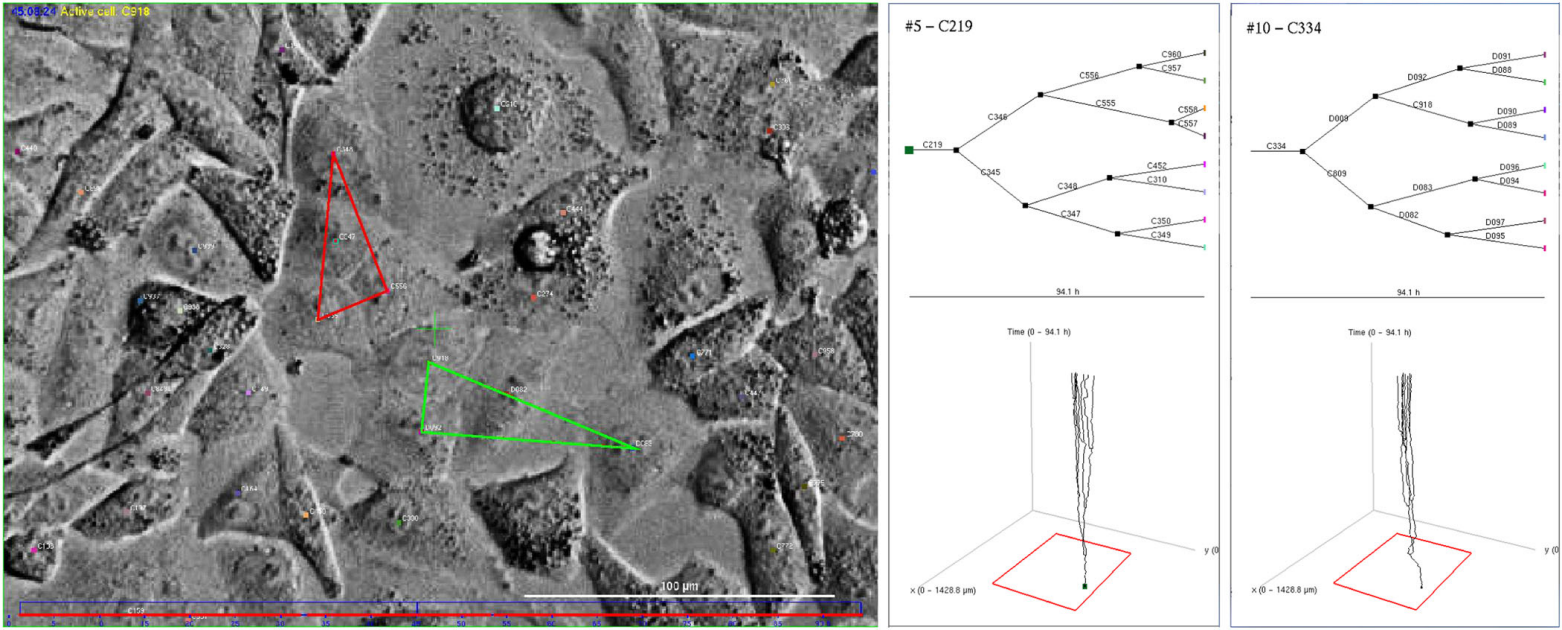
Visual illustration of morphological similarities between cells in the same lineage. This is an argument that combined analysis of cells in pedigree trees can provide more information as compared to analysis of cells without knowing their close relatives. Left: Snapshot from video of A549 cells after 45 h expossure to yessotoxin. The middle and right sections depict pedigree trees, with the lower portion demonstrating the movement of cells in the above pedigree tree during recording. The time axis is represented upwards. The red triangle points out cells in the pedigree tree with root cell C219 (middle of the figure) whereas the green triangle points out cells in the pedigree tree with root cell C334 (right part of the figure). Note that these cells form clusters.

Figure 7 supports the notion of signaling downstream lineages by demonstrating visual evidence of morphological similarities among cells within the same pedigree tree, in contrast to the surrounding cells. Moreover, the corresponding pedigree trees and movements also exhibit resemblances. These observations strongly imply that establishing connections between cells in pedigree trees can significantly aid the analysis of single cells. Classification of cells, for example, often involves a certain level of uncertainty. However, by adopting a combined classification approach specifically designed for pedigree trees, it can be feasible to mitigate this inherent uncertainty.

### 3.5 Mean square displacement (MSD) of first generation daughter cells

The MSD of cells over time is a measure that captures both their speed and directional persistence. Statistics from it can presumably help big data analyses to find causal relations in large sets of single‐cell tracking data. Such data can also have direct interest in special studies. [46] for example, argue for the importance of acquiring such data for better understanding tumor growth rate and size.

**Figure 8:**
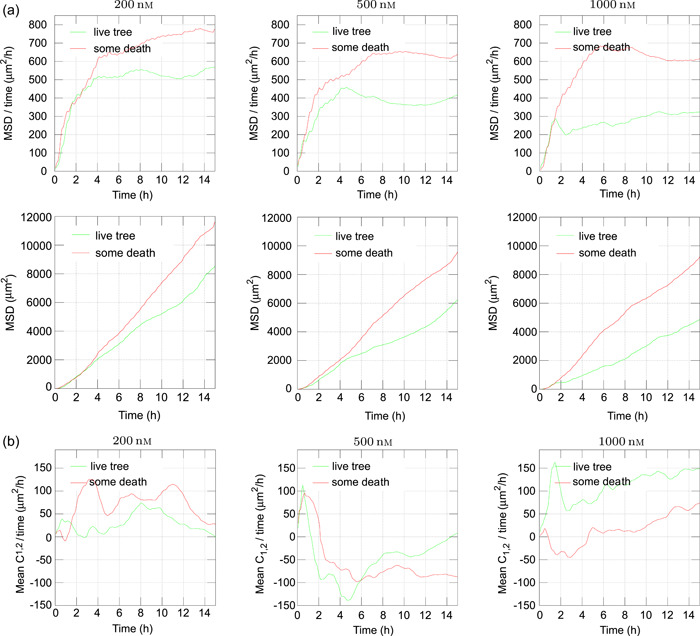
Examples of statistics of displacements of sister cells after their birth. The cells are subject to exposure by Yessotoxin (YTX) at concentrations of 200, 500, and 1000 nM. (a) Upper row: Mean square displacement (MSD) of individual first‐generation daughter cells (i.e., ***c*** (*t*)/*t*, see Equation 2) as a function of time *t* from their birth (divided by *t*) Lower row: MSD of first generation daughter cells as a function of time from their birth. “live tree”: for cells in pedigree trees with no cell death (during recording period). “some death”: for cells in pedigree trees with some cell death (during recording period). (b) Average values of ***c***_1,2_(*t*)/*t* (see Equation 3) for cells in pedigree trees with and without cell death 200, 500, and 1000 nM.

This section explores potential methods for extracting features from such data, aligning with the principles discussed in Sections 3.2, 3.3, and 3.4. Figure 8a illustrates the MSD of first‐generation daughter cells, depicting their displacement as a function of time since birth. The figure is for cells in pedigree trees, with and without cell death during recording. The MSD of cells over time reflects both their speed and movement patterns. This section explores potential methods for extracting features from such data, aligning with the principles discussed in Sections 3.2, 3.3, and 3.4. Figure 8a illustrates the MSD of first‐generation daughter cells, depicting their displacement as a function of time since birth. The upper row here shows the tendency of cells to need extra time to start drifting from their place of birth. Processing of more data may reveal if this extra time can be considered a “phenotype” useful for search in data from many diverse experiments.

Note that Figure 8a indicates that cells in lineages with dying cells tend to move faster from their initial position as compared to cells with no observed cell death. A possible hypothesis is that cells with the strongest (inheritable) tendencies to move, are more vulnerable to the actual toxin (YTX) as compared to the others. One may also relate the observation to the concept of “fight‐or‐flight” reaction, where many types of cells respond to a variety of stressors in a reasonably standardized fashion, which allows them to combat the offending stimulus or escape from it [49].

If the movement follows a “memory‐less” Brownian‐type motion, the graphs for the upper row in Figure 8a would appear as straight horizontal lines, while the lower row would exhibit straight upward tilting lines. However, the actual graphs of Figure 8a reflect that the direction of movement tends to be independent of the direction about 4–6 h earlier. The period up to about 4 h is “memory time” reflecting how long cells tend to keep their direction. It can partly correlate with cell shape, assuming elongated cells move in their longitudinal direction.

Assume the vector ***r*** (*t*) represents the relocation of a cell *t* time units after its birth. The vector dot (inner) product

(2) c(t)=r(t)·r(t),

then gives this distance squared (equal | ***r*** (*t*)|^2^). Figure 8a shows average values for *c*(*t*) for two subsets of cells where *t* ranges from 0 to 15 h. A tempting idea is slightly to modify this elaboration and check for an average value of

(3) $$c_{1,2}\left(t\right)=r_{1}\left(t\right)\;\cdot \;r_{2}\left(t\right),$$

where ***r***_1_ (*t*) and ***r***_2_ (*t*) each represent the positions of a couple of siblings (sister cells) *t* time units after their birth. Figure 8b shows an example of results from such a numerical experiment. The motivation for this test is the conceptual simplicity and pure formal similarity between Equations (2) and (3). The authors have no specific biological interpretations of these graphs, except that they reflect the tendency for sister cells to follow each other after their birth. This tendency seems to depend on exposure.

### 3.6 Material exchange and trait inheritance

**Figure 9:**
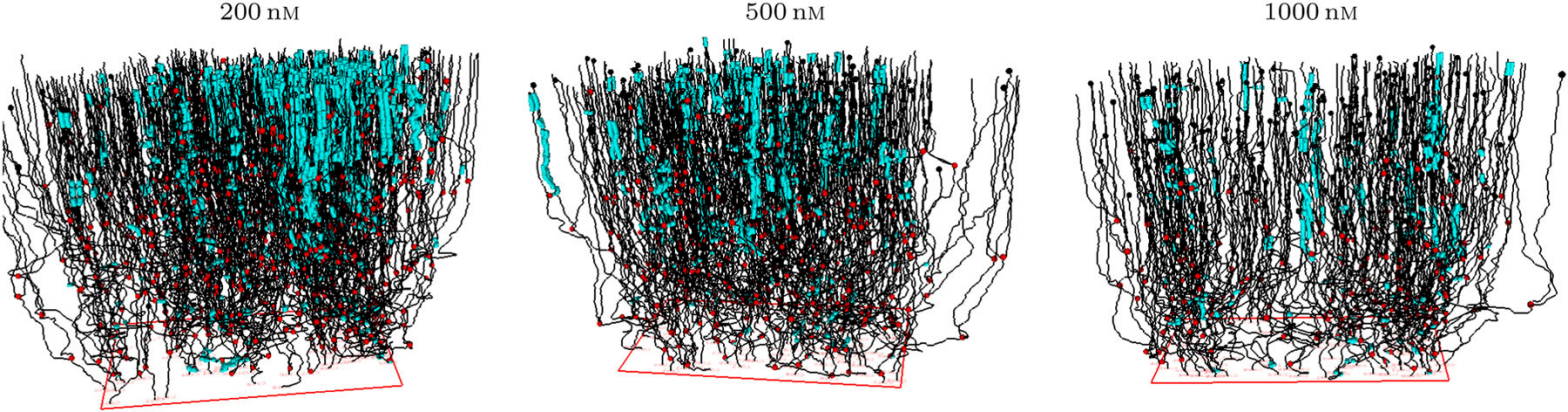
Forest of pedigree trees including identification of events where cells stay at the vicinity of each other for at least 4 h 2 h apart from their birth (cell division). The cells were subject to Yessotoxin (YTX) exposure at concentrations of 200, 500, and 1000 nM.

Moving cells are capable of maintaining close proximity for extended periods, which may suggest intercellular communication or material exchange that can impact their behavior. The specific characterization of this behavior is a subject for future research. Figure 9 exemplifies the identification of these events where cells exhibit prolonged closeness. This type of data may have special interest for coculture or studies on differentiation where interactions are crucial. Cells can interact through physical contact, surface receptor‐ligand interaction, cellular junctions, and secreted stimulus [30]. Understanding these types of interactions can contribute to deciphering the complex network of interaction between cells, helping to improve therapeutics [30].

**Figure 10:**
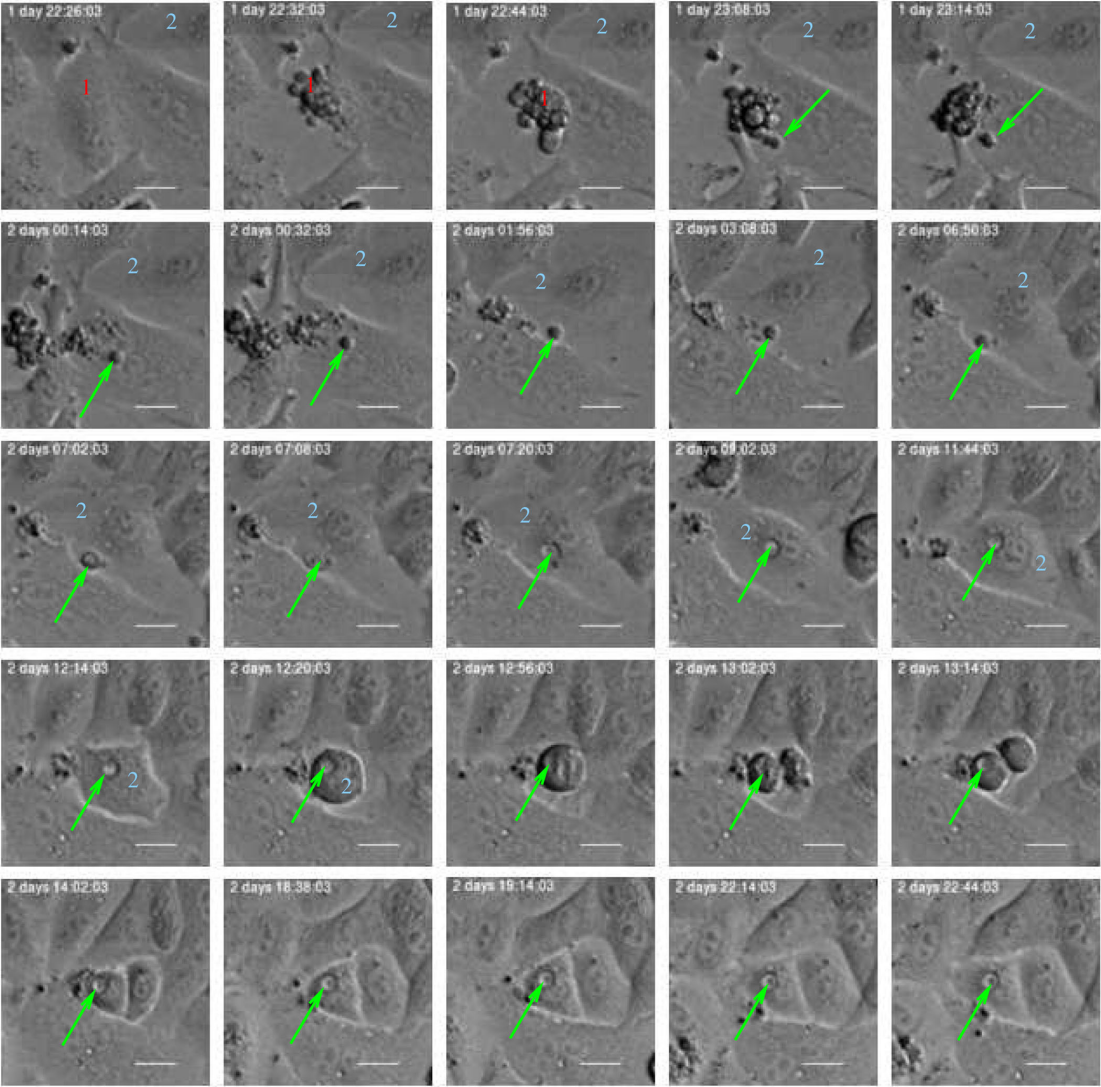
Example where an apoptotic body (green arrow) from a dying cell (1) ends up as a vacuole in a neighboring cell (2) which subsequently divides, and the vacuole ends up in one of the daughter cells. Detailed inspection of many cells in video can reveal such rare events and shed light on epigenetic heritage, and generally signaling downstream pedigree trees. Such signaling is an argument to study lineages as independent entities and for example, apply information on lineage relations when for example classifying cells.

Analyses of “forests” of pedigree trees can reflect effects from events where cells absorb debris from dead cells and transfer it to their descendants. Figure 10 shows an example of such behavior where a cell includes an apoptotic body from a neighboring dying cell. Such apoptotic bodies can subsequently appear as vacuoles in the absorbing cell. Sets of such vacuoles in a cell are traceable throughout cell division by comparing their size and number.

## 4 DISCUSSION

This work illustrates a number of possible methods to refine (or compress) data from video‐based single‐cell tracking. The main intention is to provide relevant input for big data analysis (or machine learning in general) to identify biomarkers for better diagnosis and prognosis. Well‐proven fault‐tolerant computerized methods are here available to search for causal relations in large data sets [50, 51, 52]. The principle of Occam's razor [53, 54] can guide the search, favouring simplifications and approximations. It can be considered a contradiction to anticipate the exact result from trying big data analysis methods, nor can one expect to anticipate which refinement methods are most effective. Successful big data analysis is (similar to data mining) assumed beyond the reach of human brains. However, their result may finally be understood by humans.

Big data methods go beyond assuming linear association between variables. The present examples therefore restrict to visual/intuitive illustrations of data refinement left for further processing. The existence of several local maxima in joint distributions (clustering) may, as an example, reflect significant biological information. The left part of Figure 4 illustrates this point. It shows two main maxima of the joint distribution of number of descendants of sister cells. This may indicate inheritance of robustness/viability, making it likely for the most robust cells finally to dominate in number (which could be relevant for prognoses in cancer).

The present examples of refinement methods typically show different behavior of cells in pedigree trees with cell death as compared to the behavior of cells in pedigree trees without cell death (during recording). Some of these examples also show correlations between sister cells or descendants of sister cells. This is an argument to treat whole pedigree trees as individual entities in the initial data refinement.

Successful application of big data analysis can, in addition to sort out causal relations, give the possibility to search for similarities between the behavior of cells in many experiments. Methods to compare experiments can in general be an important part of a collective knowledge base of cell behavior.

Recent progress in techniques for sparse representations, compressive sensing, and machine learning (see e.g., [55, 56]) give a perspective of direct automatic identification of actual biomarkers directly from video of cells. The present work contributes to this development by demonstrating initial refinement of data from single‐cell tracking. These data summaries may also be of direct biological or medical interest in the conceptual framework of standalone experiments. They may in addition help the development of formal mathematical methods by applying concepts from statistical physics [57]. However, note that machine search for causality in data may utilize weak correlations without any immediate intuitive meaning.

This work illustrates derivation of the following parameters from single‐cell tracking data which represent positions of individual live cells, their division, and death during several cell cycles:

- Number of cells in different classes of pedigree trees during video recording (Section 3.2). It may reflect that some pedigree trees consist of specially viable and resilient cells. This property seems to be already written into the root (ancestor) cell. Intrusive single‐cell analysis after tracking, while preserving track identities, may clarify the corresponding mechanisms behind this resilience.
- Parameters from (representations of) speed distributions for various subsets of cells during tracking (Figure 5). The regularity of these distributions allow representations by few parameters (so‐called sparse representation).
- Parameters from joint distributions of the size of (pedigree) subtrees for the first generation sister cells where they are root cells (Figure 6). Such distributions can be parameterized by correlation coefficients, covariance, and shape parameters (or sparse representations).
- “Memory” time of trajectories for cells in subpopulations. Figure 8a reveals that cell trajectories can have a tendency to keep their direction, typically during 2 h to 4 h. This tendency can reflect cell shape.
- Tendency for cells to stay close to each other for periods. Figure 9 visualizes an example where cells tend to stay close for periods of time. Such events can potentially reflect intercellular communication and material exchange (see Figure 10). This tendency may have a special interest in studies where communication between different cell types plays a role. Tracking of cells in coculture can in this case help to reveal how to affect such behavior.

An intention behind the present work is, as pointed out above, to promote ideas for better and easier comparison between different experiments. This would promote securing reproducibility of observations, which has emerged as a main concern in life science research in recent years [58]. Easy exchange of raw and refined data is paramount in such quality assurance. Experiments on cells can include video recording of them under standard (common) conditions, and statistics from tracking the cells can reveal differences between experiments and which can affect their reproducibility. Tracking under standard conditions may in general reveal effects on cells and which otherwise may pass under the radar using bulk assays. This is an example of direct use of the present type of statistics.

Large‐scale sharing of data from tracking single cells in video will naturally raise questions on robustness of results from initial analysis of them. Cells in different experiments may never be treated exactly the same way. Cells can be sensitive to photo‐toxicity as well as possible molecular probes. Types of extracellular matrices and their proteins can also affect cellular behavior in test wells [59]. Data analysis can reveal to what degree comparisons of data from them still apply. It will be important to identify ranges of conditions for cells in which they will behave in comparable ways. It will also be important to identify conditions/treatments where cellular behavior is sensitive to small and uncontrollable perturbations. Data analysis may also reveal possible probabilistic views of results from observing cellular behavior.

Further development of sensors and software will extend the above restriction to data on cell positions, division, and death. This will advance exploitation of its potential utility, as indicated by several authors [16, 23, 60, 61, 62]. Single‐cell tracking from high‐quality imagery allows collecting data on phenotypical changes, otherwise difficult to measure from an end‐point measurement such as single‐cell RNA‐sequencing (scRNA‐seq) [62]. Furthermore, epigenetic states, protein expression, and enzyme activity, can not only be inferred from changes in gene expression [62, 63]. Integrating single‐cell tracking with RNA‐seq analysis can therefore complement characterization of biological processes by combining analysis of cellular phenotypes with gene expression profiles [64, 65]. These analyses allow overlaying phenotypic cell identity with genetic lineage information for a more comprehensive view of clonal relationships, since gene expression alone is not sufficient to classify cell states [4, 66]. Integrating such analysis into cell ontology can help to discover a large variety of novel cell populations [45]. Tracking individual cells can therefore complement current cell ontology efforts.

Big data analysis relies on a significant amount of data to derive meaningful insights, and accurately assessing the value of a data set is only possible once it becomes available for analysis. As a result, the authors assert that a comprehensive roadmap for substantial and meaningful data sharing should involve the prototyping of statistical parameters and the creation of value through the execution of complementary specialized studies. The authors' current contribution aims to serve as an inspiration for such specialized studies, motivating researchers to delve further into this field of research.

## AUTHOR CONTRIBUTIONS

**Mónica Suárez Korsnes**: Conceptualization (equal); data curation (equal); formal analysis (equal); funding acquisition (equal); investigation (equal); methodology (equal); project administration (equal); resources (equal); software (supporting); supervision (equal); validation (equal); visualization (equal); writing—original draft (equal); writing—review and editing (equal). **Reinert Korsnes**: Conceptualization (equal); data curation (equal); formal analysis (lead); funding acquisition (equal); investigation (equal); methodology (equal); project administration (equal); resources (equal); software (lead); supervision (equal); validation (equal); visualization (equal); writing—original draft (equal); writing—review and editing (equal).

## CONFLICT OF INTEREST STATEMENT

Mónica Suárez Korsnes is the owner of the upstart firm Korsnes Biocomputing (KoBio) aimed to participate in research and development of methods for single‐cell analysis. The remaining author declares no conflict of interest.

5

## ETHICS STATEMENT

Not applicable.

## INFORMED CONSENT

Not applicable.

## Data Availability

The data used to support the findings of this study are available from the corresponding authors upon request.

## Footnotes

^1^

https://www.korsnesbiocomputing.no/

^2^
Supplementary data are available via https://user.korsnesbiocomputing.no (user inref 2022, password korsnes1)

^3^

https://www.korsnesbiocomputing.no/

^4^

http://www.scholarpedia.org/article/Mutual_information

^5^

https://scipy.org/

## References

[1] Skylaki S , Hilsenbeck O , Schroeder T . Challenges in long‐term imaging and quantification of single‐cell dynamics. Nat Biotechnol. 2016;34(11):1137–44. 10.1038/nbt.3713 27824848

[2] Tata PR , Rajagopal J . Cellular plasticity: 1712 to the present day. Curr Opin Cell Biol. 2016;43:46–54. 10.1016/j.ceb.2016.07.005 27485353 PMC5154913

[3] Gupta PB , Pastushenko I , Skibinski A , Blanpain C , Kuperwasser C . Phenotypic plasticity: driver of cancer initiation, progression, and therapy resistance. Cell Stem Cell. 2019;24(1):65–78. 10.1016/j.stem.2018.11.011 30554963 PMC7297507

[4] Woodworth MB , Girskis KM , Walsh CA . Building a lineage from single cells: genetic techniques for cell lineage tracking. Nat Rev Genet. 2017;18(4):230–44. 10.1038/nrg.2016.159 28111472 PMC5459401

[5] Lüönd F , Tiede S , Christofori G . Breast cancer as an example of tumour heterogeneity and tumour cell plasticity during malignant progression. Br J Cancer. 2021;125(2):164–75. 10.1038/s41416-021-01328-7 33824479 PMC8292450

[6] Melzer C , von der Ohe J , Hass R . Enhanced metastatic capacity of breast cancer cells after interaction and hybrid formation with mesenchymal stroma/stem cells (MSC). Cell Commun Signal. 2018;16(1):2. 10.1186/s12964-018-0215-4 29329589 PMC5795285

[7] Shabo I , Svanvik J , Lindström A , Lechertier T , Trabulo S , Hulit J , et al. Roles of cell fusion, hybridization and polyploid cell formation in cancer metastasis. World J Clin Oncol. 2020;11(3):121–35. 10.5306/wjco.v11.i3.121 32257843 PMC7103524

[8] Hass R , von der Ohe J , Ungefroren H . Impact of the tumor microenvironment on tumor heterogeneity and consequences for cancer cell plasticity and stemness. Cancers. 2020;12(12):3716. 10.3390/cancers12123716 33322354 PMC7764513

[9] Chang HH , Hemberg M , Barahona M , Ingber DE , Huang S . Transcriptome‐ wide noise controls lineage choice in mammalian progenitor cells. Nature. 2008;453(7194):544–7. 10.1038/nature06965 18497826 PMC5546414

[10] Wakita S , Yamaguchi H , Omori I , Terada K , Ueda T , Manabe E , et al. Mutations of the epigenetics‐modifying gene (DNMT3a, TET2, IDH1/2) at diagnosis may induce FLT3‐ITD at relapse in de novo acute myeloid leukemia. Leukemia. 2013;27(5):1044–52. 10.1038/leu.2012.317 23135354

[11] Bheda P , Schneider R . Epigenetics reloaded: the single‐cell revolution. Trends Cell Biol. 2014;24(11):712–23. 10.1016/j.tcb.2014.08.010 25283892

[12] Shinjo K , Kondo Y . Targeting cancer epigenetics: linking basic biology to clinical medicine. Adv Drug Deliv Rev. 2015;95:56–64. 10.1016/j.addr.2015.10.006 26494398

[13] Bintu L , Yong J , Antebi YE , McCue K , Kazuki Y , Uno N , et al. Dynamics of epigenetic regulation at the single‐cell level. Science. 2016;351(6274):720–4. 10.1126/science.aab2956 26912859 PMC5108652

[14] Regot S , Hughey JJ , Bajar BT , Carrasco S , Covert MW . High‐sensitivity measurements of multiple kinase activities in live single cells. Cell. 2014;157(7):1724–34. 10.1016/j.cell.2014.04.039 24949979 PMC4097317

[15] Suman R , Smith G , Hazel KEA , Kasprowicz R , Coles M , O'Toole P , et al. Label‐free imaging to study phenotypic behavioural traits of cells in complex co‐cultures. Sci Rep. 2016;6(1):22032. 10.1038/srep22032 26915695 PMC4768090

[16] Van Valen DA , Kudo T , Lane KM , Macklin DN , Quach NT , DeFelice MM , et al. Deep learning automates the quantitative analysis of individual cells in live‐cell imaging experiments. PLoS Comput Biol. 2016;12(11):e1005177. 10.1371/journal.pcbi.1005177 27814364 PMC5096676

[17] Koh SB , Mascalchi P , Rodriguez E , Lin Y , Jodrell DI , Richards FM , et al. A quantitative FastFUCCI assay defines cell cycle dynamics at a single‐cell level. J Cell Sci. 2017;130(2):512–20. 10.1242/jcs.195164 27888217

[18] DuChez BJ . Automated tracking of cell migration with rapid data analysis. Curr Protocols Cell Biol. 2018;76:12.12.1–16. 10.1002/cpcb.28 PMC5654555 28862338

[19] Korsnes MS , Korsnes R . Single‐cell tracking of a549 lung cancer cells exposed to a marine toxin reveals correlations in pedigree tree proﬁles. Front Oncol. 2018;8:260. 10.3389/fonc.2018.00260 30023341 PMC6039982

[20] Emami N , Sedaei Z , Ferdousi R . Computerized cell tracking: current methods, tools and challenges. Visual Inform. 2021;5(1):1–13. 10.1016/j.visinf.2020.11.003

[21] Fujimoto K , Seno S , Shigeta H , Mashita T , Ishii M , Matsuda H . Tracking and analysis of fucci‐labeled cells based on particle ﬁlters and time‐to‐event analysis. IJBBB. 2020;10(2):94–109. 10.17706/ijbbb.2020.10.2.94-109

[22] Fazeli E , Roy NH , Follain G , Laine RF , von Chamier L , Hänninen PE , et al. Automated cell tracking using stardist and trackmate. F1000Res. 2020;9:1279. 10.12688/f1000research.27019.1 33224481 PMC7670479

[23] Ghannoum S , Antos K , Leoncio Netto W , Gomes C , Köhn‐Luque A , Farhan H , et al. Cellmaptracer: a user‐friendly tracking tool for long‐term migratory and proliferating cells associated with Fucci systems. Cells. 2021;10(2):469. 10.3390/cells10020469 33671785 PMC7927118

[24] Korsnes MS , Korsnes R . Lifetime distributions from tracking individual bc3h1 cells subjected to yessotoxin. Front Bioeng Biotechnol. 2015;3:166. 10.3389/fbioe.2015.00166 26557641 PMC4617161

[25] Korsnes MS , Korsnes R . Mitotic catastrophe in bc3h1 cells following yessotoxin exposure. Front Cell Dev Biol. 2017;5:30. 10.3389/fcell.2017.00030 28409150 PMC5374163

[26] Korsnes MS , Kolstad H , Kleiveland CR , Korsnes R , Ørmen E . Autophagic activity in BC3H1 cells exposed to yessotoxin. Toxicol In Vitro. 2016;32:166–80. 10.1016/j.tiv.2015.12.010 26743762

[27] Klionsky DJ , Abdel‐Aziz AK , Abdelfatah S , Abdellatif M , Abdoli A , Abel S , et al. Guidelines for the use and interpretation of assays for monitoring. Autophagy. 2021;17(1):1–382. 10.1080/15548627.2020.1797280 33634751 PMC7996087

[28] Andrei L , Kasas S , Ochoa Garrido I , Stanković T , Suárez Korsnes M , Va‐ clavikova R , et al. Advanced technological tools to study multidrug resistance in cancer. Drug Resist Updates. 2020;48:100658. 10.1016/j.drup.2019.100658 31678863

[29] Langhans SA . Three‐dimensional *in vitro* cell culture models in drug discovery and drug repositioning. Front Pharmacol. 2018;9:6. 10.3389/fphar.2018.00006 29410625 PMC5787088

[30] Nishida‐Aoki N , Gujral TS . Emerging approaches to study cell–cell interactions in tumor microenvironment. Oncotarget. 2019;10(7):785–97. 10.18632/oncotarget.26585 30774780 PMC6366828

[31] Edlund C , Jackson TR , Khalid N , Bevan N , Dale T , Dengel A , et al. Livecell‐A large‐scale dataset for label‐free live cell segmentation. Nat Methods. 2021;18(9):1038–45. 10.1038/s41592-021-01249-6 34462594 PMC8440198

[32] Helgadottir S , Midtvedt B , Pineda J , Sabirsh A , Adiels C. B. , Romeo S , et al. Extracting quantitative biological information from bright‐field cell images using deep learning. Biophys Rev. 2021;2(3):031401. 10.1063/5.0044782 PMC10903417 38505631

[33] Capuzzo AM , Vigo D . Microﬂuidic live‐imaging technology to perform research activities in 3D models. Ann Adv Biomed Sci. 2021;4(1):1–38. 10.1101/2021.03.08.434339

[34] Aw Yong KM , Li Z , Merajver SD , Fu J . Tracking the tumor invasion front using long‐term ﬂuidic tumoroid culture. Sci Reports. 2017;7(1):1–7. 10.1038/s41598-017-10874-1 PMC5589910 28883652

[35] Finnberg NK , Gokare P , Lev A , Grivennikov SI , MacFarlane AW , Campbell KS , et al. Application of 3D tumoroid systems to define immune and cytotoxic therapeutic responses based on tumoroid and tissue slice culture molecular signatures. Oncotarget. 2017;8(40):66747–57. 10.18632/oncotarget.19965 28977993 PMC5620133

[36] Puls TJ , Tan X , Whittington CF , Voytik‐Harbin SL . 3D collagen ﬁbrillar microstructure guides pancreatic cancer cell phenotype and serves as a critical design parameter for phenotypic models of emt. PLoS ONE. 2017;12(11):e0188870. 10.1371/journal.pone.0188870 29190794 PMC5708668

[37] Fontana F , Marzagalli M , Sommariva M , Gagliano N , Limonta P . In vitro 3d cultures to model the tumor microenvironment. Cancers. 2021;13(12):2970. 10.3390/cancers13122970 34199324 PMC8231786

[38] Wen C , Miura T , Voleti V , Yamaguchi K , Tsutsumi M , Yamamoto K , et al. 3DeeCellTracker, a deep learning‐based pipeline for segmenting and tracking cells in 3D time‐lapse images. eLife. 2021;10:e59187. 10.7554/eLife.59187 33781383 PMC8009680

[39] Schmitz J , Täuber S , Westerwalbesloh C , Lieres E , Noll T , Grünberger A . Development and application of a cultivation platform for mammalian suspension cell lines with single‐cell resolution. Biotechnol Bioeng. 2021;118(2):992–1005. 10.1002/bit.27627 33200818

[40] Schmitz J , Stute B , Tauber S , Kohlheyer D , von Lieres E , Grunberger A . Reliable cell retention of mammalian suspension cells in microﬂuidic cultivation chambers. Sci Rep. 2023;13(1):3857. 10.1038/s41598-023-30297-5 36890160 PMC9995442

[41] Korsnes MS . Yessotoxin as a tool to study induction of multiple cell death pathways. Toxins. 2012;4(7):568–79. 10.3390/toxins4070568 22852069 PMC3407893

[42] Tinevez JY , Perry N , Schindelin J , Hoopes GM , Reynolds GD , Laplantine E , et al. TrackMate: An open and extensible platform for single‐particle tracking. Methods. 2017;115:80–90. 10.1016/j.ymeth.2016.09.016 27713081

[43] Zou D , Ma L , Yu J , Zhang Z . Biological databases for human research. Genom Insights. 2015;13(1):55–63. 10.1016/j.gpb.2015.01.006 PMC4411498 25712261

[44] Haniffa M , Taylor D , Linnarsson S , Aronow BJ , Bader GD , Barker RA , et al. A roadmap for the human developmental cell Atlas. Nature. 2021;597(7875):196–205. 10.1038/s41586-021-03620-1 34497388 PMC10337595

[45] Osumi‐Sutherland D , Xu C , Keays M , Kharchenko PV , Regev A , Lein E , et al. Cell types and ontologies of the human cell atlas. arXiv preprint arXiv:2106. 14443, 2021. 10.1038/s41556-021-00787-7 34750578

[46] Ghannoum S , Fantini D , Zahoor M , Reiterer V , Phuyal S , Leon‐ cio Netto W , et al. A combined experimental‐computational approach uncovers a role for the Golgi matrix protein giantin in breast cancer progression. PLoS Comput Biol. 2023;19(4):e1010995. 10.1371/journal.pcbi.1010995 37068117 PMC10159355

[47] Fodor IK . A survey of dimension reduction techniques. Lawrence Livermore National Lab; 2002.

[48] Wand MP , Jones MC . Kernel smoothing. CRC Press; 1994.

[49] Goligorsky MS . The concept of cellular “ﬁght‐or‐ﬂight” reaction to stress. Am J Physiol Renal Physiol. 2001;280(4):F551–61. 10.1152/ajprenal.2001.280.4.F551 11249846

[50] Theodoridis S . Machine learning: a Bayesian and optimization perspective, Academic Press; 2015.

[51] Kotsiantis S.B , Zaharakis I , Pintelas P , et al. Supervised machine learning: a review of classiﬁcation techniques. Emerg Arti Intell Appl Comp Eng. 2007;31(3):249–68.

[52] Linero AR . A review of tree‐based Bayesian methods. Commun Statist Appl Methods. 2017;24(6):543–59. 10.29220/CSAM.2017.24.6.543

[53] Domingos P . Occam's two razors: The sharp and the blunt. In *Proceedings of the Fourth International Conference on Knowledge Discovery and Data Mining (KDD'98)*. AAAI Press; 1998:37–43. https://dl.acm.org/doi/10.5555/3000292.3000299

[54] Domingos P . The role of Occam's Razor in knowledge discovery. Data Mining Knowl Disc. 1999;3(4):409–25. 10.1023/A:1009868929893

[55] Papyan V , Romano Y , Sulam J , Elad M . Theoretical foundations of deep learning via sparse representations: a multilayer sparse model and its connection to convolutional neural networks. IEEE Signal Process Magaz. 2018;35(4):72–89. 10.1109/MSP.2018.2820224

[56] Mousavi A , Rezaee M , Ayanzadeh R . A survey on compressive sensing: Classical results and recent advancements. arXiv preprint arXiv:1908. 01014v3, 2019. 10.48550/arXiv.1908.01014

[57] Banigan EJ . Statistical physical models of cellular motility. University of Pennsylvania; 2013.

[58] Hirsch C , Schildknecht S . *In vitro* research reproducibility: keeping up high standards. Front Pharmacol. 2019;10:1484. 10.3389/fphar.2019.01484 31920667 PMC6916005

[59] Vigilante A , Laddach A , Moens N , Meleckyte R , Leha A , Ghahramani A , et al. Identifying extrinsic versus intrinsic drivers of variation in cell behavior in human iPSC lines from healthy donors. Cell Rep. 2019;26(8):2078–87. 10.1016/j.celrep.2019.01.094 30784590 PMC6381787

[60] Tsai HF , Gajda J , Sloan TFW , Rares A , Shen AQ . Usiigaci: instance‐aware cell tracking in stain‐free phase contrast microscopy enabled by machine learning. SoftwareX. 2019;9:230–7. 10.1016/j.softx.2019.02.007

[61] Moen E , Borba E , Miller G , Schwartz M , Bannon D , Koe N , et al. Accurate cell tracking and lineage construction in live‐cell imaging experiments with deep learning. bioRxiv, 803205; 2019. 10.1101/803205

[62] Liu Z , Yuan J , Lasorella A , Iavarone A , Bruce JN , Canoll P , et al. Integrating single‐cell rna‐seq and imaging with scope‐seq2. Sci Rep. 2020;10(1):19482. 10.1038/s41598-020-76599-w 33173156 PMC7655825

[63] Zhang JQ , Siltanen CA , Liu L , Chang KC , Gartner ZJ , Abate AR . Linked optical and gene expression proﬁling of single cells at high‐throughput. Genome Biol. 2020;21(1):1–11. 10.1186/s13059-020-01958-9 PMC7041248 32093753

[64] Lane K , Van Valen D , DeFelice MM , Macklin DN , Kudo T , Jaimovich A , et al. Measuring signaling and RNA‐seq in the same cell links gene expression to dynamic patterns of NF‐κb activation. Cell Syst. 2017;4(4):458–69. 10.1016/j.cels.2017.03.010 28396000 PMC6748049

[65] Yuan J , Sheng J , Sims PA . Scope‐seq: a scalable technology for linking live cell imaging and single‐cell RNA sequencing. Genome Biol. 2018;19(1):227. 10.1186/s13059-018-1607-x 30583733 PMC6305572

[66] Gerbin KA , Grancharova T , Donovan‐Maiye RM , Hendershott MC , Anderson HG , Brown JM , et al. Cell states beyond transcriptomics: integrating structural organization and gene expression in hipsc‐derived cardiomyocytes. Cell Systems. 2021;12(6):670–87. 10.1016/j.cels.2021.05.001 34043964

